# *RASSF1A*基因甲基化与非小细胞肺癌预后的相关性研究

**DOI:** 10.3779/j.issn.1009-3419.2010.04.08

**Published:** 2010-04-20

**Authors:** 卉 张, 树才 张, 宗德 张, 红彦 贾, 淑香 古, 丹 赵

**Affiliations:** 101149 北京，北京胸科医院肿瘤内科 Department of Medical Oncology, Beijing Chest Hospital, Beijing 101149, China

**Keywords:** 肺肿瘤, 甲基化, 预后, Lung neoplasms, Methylation, Prognosis

## Abstract

**背景与目的:**

研究发现在很多肿瘤中都存在*RASSF1A*基因启动子区域高甲基化状态导致基因表达失活的现象，本研究就*RASSF1A*基因启动子的甲基化状态与非小细胞肺癌预后的关系进行探讨。

**方法:**

采用甲基化特异的PCR检测150例非小细胞肺癌和20例肺部良性病变RASSF1A启动子甲基化状态。

**结果:**

150例非小细胞肺癌中58例发现RASSF1A启动子存在甲基化（58/158, 38.7%），20例肺部良性病变中无一例发现RASSF1A启动子甲基化。存在RASSF1A启动子高甲基化的病例预后较未发现RASSF1A甲基化的病例差（*P*=0.004），*Cox*回归分析显示RASSF1A启动子的甲基化状态是非小细胞肺癌术后的一个预后相关因素（RR=1.584, 95%CI: 1.040-2.411, *P*=0.032）。

**结论:**

MSP法检测RASSF1A启动子甲基化状态可以作为非小细胞肺癌术后的一个预后评价指标。

## Abstract (en)

**Background and objective:**

Promoter hypermethylation of the *RASSF1A* gene is among the most abundant epigenetic deregulations in human cancer. The aim of this study is to investigate the relationship between the methylation status of RASSF1A promoter and the prognoses of non-small cell lung cancer (NSCLC).

**Methods:**

The methylation status of RASSF1A promoter in 150 NSCLC and 25 non-malignant tissues was determined using a methylation-specific polymerase chain reaction (MSP).

**Results:**

RASSF1A promoter hypermethylation was detected in 38.7% (58/150) of NSCLC tissues, but in none of the non-malignant tissues. The patients with hypermethylation of RASSF1A had a poor survival rate, and the relationship between the survival rate and hypermethylation of RASSF1A was statistically significant (*P*=0.004). Then by using stepwise *Cox* proportional hazard regression testing, methylation status of RASSF1A was an independent factor affecting the NSCLC patients' survival (RR=1.584, 95%CI: 1.040-2.411, *P*=0.032).

**Conclusion:**

The hypermethylation of the RASSF1A promoter may be an independent prognostic factor of NSCLC after operation.

近年来随着表观遗传学的发展，研究发现DNA甲基化在真核生物基因表达调控中起着重要的作用。在肺癌组织中，已经发现一系列抑癌基因存在启动子区异常高甲基化所导致的失活现象。其中部分抑癌基因的高甲基化与肿瘤发生、病情进展和预后密切相关，提示基因的甲基化可能是肺癌诊断、治疗和预后评价的生物学标志之一。

自2000年Dammann等^[1]^从人染色体3p21.3区域分离鉴定出*RASSF1A*并确定其为一新型抑癌基因至今，已有多篇文献报道RASSF1A启动子区高甲基化与肺癌患者预后相关。国内亦有关于RASSF1A在肿瘤组织中表达异常的报道，但与肺癌预后相关性报道较少，因此我们就非小细胞肺癌（non-small cell lung cancer, NSCLC）中RASSF1A异常甲基化与患者生存预后进行了临床研究。

## 1 材料与方法

### 1.1 材料

所取病例标本为1999年-2000年北京胸部肿瘤结核病医院胸外科进行手术切除的150例NSCLC组织和20例肺部良性病变组织。手术切除组织标本即刻-70 ℃低温保存。每例肿瘤组织标本均经高倍境下确定肿瘤细胞≥80%。所有病例术前均未经放疗和化疗。150例NSCLC病例中男性121例，女性29例；年龄为31岁-78岁，平均年龄为（59±9）岁；其中腺癌40例，鳞癌102例，腺鳞癌8例；根据世界卫生组织（WHO）的标准：肿瘤TNM分期为Ⅰ期49例，Ⅱ期32例，Ⅲa期48例，Ⅲb-Ⅳ期21例，肿瘤分化程度为低分化62例，中分化58例，高分化30例。

### 1.2 DNA提取酚/氯仿抽提法。

### 1.3 DNA的亚硫酸氢盐修饰

取1 μg-2 μg基因组DNA，加双蒸水至50 μL，加入5 μL 3 mol/L的NaOH 37 ℃变性30 min；加入520 μL新配制的3 mol/L亚硫酸氢钠（pH5.0）和30 μL 10 mmol/L的对苯二酚，50 ℃保温16 h；纯化回收DNA（Qiaquick gel extraction kit）。

### 1.4 巢式甲基化特异性的PCR（methylation-specific polymerase chain reaction, MS-PCR）

取亚硫酸氢盐修饰后DNA 1 μL为模板，以经Sss I酶修饰的Hep-2细胞DNA为阳性对照，水为阴性对照。引物由北京赛百盛生物公司合成并纯化（表 1）。采用沉降PCR和热启动PCR。PCR反应体系为25 μL，模板DNA 1 μL，上下游引物各1 μL，DMSO 1.2 μL，2×Taq PCR Master Mix（天根生化科技有限公司）12.5 μL，最后加去离子水补齐至25 μL。第一轮扩增循环参数：94 ℃ 3 min后，94 ℃ 30 s，46 ℃-53 ℃ 50 s，72 ℃ 1 min，27次循环，72 ℃延伸10 min。第二轮扩增参数：94 ℃ 3 min后，94 ℃ 30 s，58 ℃-65 ℃ 50 s，72 ℃ 1 min，37次循环，72 ℃延伸10 min。

**1:** RASSF1A启动子区甲基化特异性PCR扩增引物序列 MSP primer sequence of RASSF1A promter

	M	U
Nest 1		
Sense primer	5’ACGAAGGAGGGAAGGAAG3’	5’GATGAAGGAGGGAAGGAA3’
Antisense primer	5’CCCGCAACTCAATAAACT3’	5’CTCCCACAACTCAATAAACT3’
Nest 2		
Sense primer	5’GGGTTTTGCGAGAGCGCG3’	5’GGTTTTGTGAGAGTGTGTTTAG3’
Antisense primer	5’GCTAACAAACGCGAACCG3’	5’CACTAACAAACACAAACCAAAC3’
Products	169 bp	169 bp
M: methylated DNA specific primer; U: unmethylated DNA specific primer.		

### 1.5 结果记录

将25 μL PCR产物进行1.5%琼脂糖凝胶电泳，然后在紫外线凝胶成像及分析系统（BIO-RAD公司）下扫描拍照，记录结果。

### 1.6 产物测序

将PCR产物进行胶回收（Qiaquick gel extraction kit），连接转化涂板，挑取阳性克隆送公司测序。

### 1.7 统计学处理

采用SPSS 13.0统计软件进行分析，相关性以χ^2^检验和*Fisher’s*精确检验进行分析；生存资料分析采用*Kaplan-Meier*法，各因素水平间比较用*Log-rank*分析，分析预后相关因素使用*Cox*回归模型，以*P* < 0.05为差异具有统计学意义。

## 2 结果

### 2.1 RASSF1A启动子区甲基化状态

150例NSCLC中58例RASSF1A启动子区高甲基化，发生甲基化率为38.7%；20例肺部良性病变中无1例RASSF1A启动子区高甲基化（图 1）。

**1:**
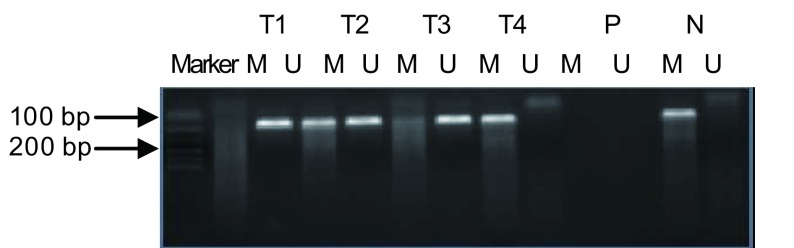
肿瘤组织、良性病变及癌旁正常组织甲基化特异性PCR产物电泳结果 Methylation analysis of RASSF1A by MSP in tissue of tumor, benign lesion and normal tissue

### 2.2 RASSF1A启动子区甲基化与临床病理因素之间的关系

RASSF1A启动子区高甲基化与年龄、性别、吸烟指数、肿瘤TNM分期均不相关，但与开始吸烟年龄、肿瘤分化程度相关。20岁前开始吸烟的NSCLC患者RASSF1A启动子甲基化率（56.7%）高于20岁后开始吸烟的NSCLC患者（28.2%）（*P*=0.006）；低分化NSCLC患者RASSF1A启动子甲基化率（51.6%）高于中分化（31.0%）和高分化（26.7%）NSCLC患者，差异有统计学意义（*P*=0.022, *P*=0.024），但中分化和高分化NSCLC之间差异无统计学意义（*P* > 0.05）（表 2）。

**2:** RASSF1A启动子甲基化与临床病理因素之间的关系 Association between methylation of RASSF1A promoter and clinicopathologic variables

Characteristic	NSCLC			Methylation rate (%)	χ^2^	*P*
	Total	Methylated	Unmethylated			
Total	150	58	92	38.7		
Age					-	> 0.05
≤60	75	29	46	38.7		
> 60	75	29	46	38.7		
Sex					1.400	> 0.05
Male	121	44	77	36.4		
Female	29	14	15	48.3		
Histology					3.408	> 0.05
Adenocarcinoma	40	18	22	45.0		
Squamous	102	35	67	34.3		
Adenosquamous	8	5	3	62.5		
Smoke index (pack-year)					2.340	> 0.05
0	42	19	23	45.2		
< 20	20	5	15	25.0		
≥20	88	34	54	38.6		
Age at starting smoking					7.608	0.006
≤20	30	17	13	56.7		
> 20	78	22	56	28.2		
Differentiated					7.628	0.022
Poorly	62	32	30	51.6		
Moderately	58	18	40	31.0		
Well	30	8	22	26.7		
TNM Stage					5.817	> 0.05
Ⅰ	49	13	36	26.5		
Ⅱ	32	12	20	37.5		
Ⅲa	48	24	24	50.0		
Ⅲb-Ⅳ	21	9	12	42.9		
NSCLC: non-small cell lung cancer.						

### 2.3 术后生存时间与RASSF1A启动子甲基化相关性

150例NSCLC术后中位生存期为33个月，58例RASSF1A启动子区高甲基化，术后中位生存期为22个月，92例未发现RASSF1A启动子区高甲基化，术后中位生存期为57个月（*P*=0.004）（图 2A）；49例Ⅰ期NSCLC术后中位生存期为80个月，13例RASSF1A启动子区甲基化，术后中位生存期为58个月，36例未发现RASSF1A启动子区高甲基化，术后中位生存期为80个月（*P*=0.022）（图 2B）；32例Ⅱ期NSCLC术后中位生存期为32个月，12例RASSF1A启动子区高甲基化，术后中位生存期为24个月，20例未发现RASSF1A启动子区高甲基化，术后中位生存期为80个月（*P*=0.023）（图 2C）；48例Ⅲa期NSCLC术后中位生存期为23个月，24例RASSFA启动子区高甲基化，术后中位生存期为18个月，24例未发现RASSF1A启动子区高甲基化，术后中位生存期为31个月（*P*=0.036）（图 2D）；21例Ⅲb-Ⅳ期NSCLC中，9例RASSF1A启动子区高甲基化，术后中位生存期为15个月，12例未发现RASSF1A启动子区高甲基化，术后中位生存期为15个月（*P*=0.118）（图 2E）。

**2:**
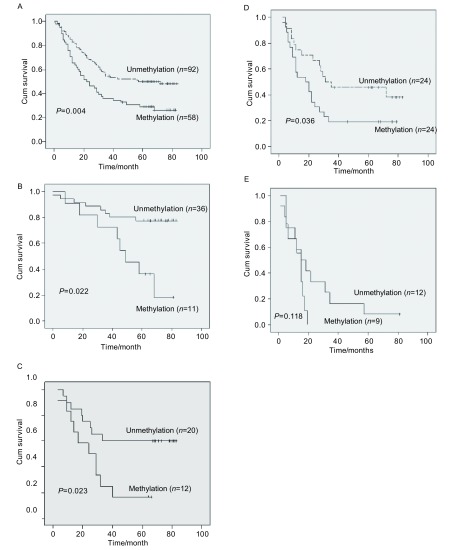
NSCLC术后RASSF1A甲基化状态相关生存曲线 Survival curves with reference to RASSF1A promoter methylation

### 2.4 RASSF1A启动子区高甲基化是NSCLC术后的一个独立预后相关因素

利用*Cox*回归模型，分析NSCLC中的各种因素（年龄、组织类型、分化程度、TNM分期、吸烟、开始吸烟年龄、甲基化状态等），发现除病理分期外RASSF1A启动子区甲基化状态也是一个与NSCLC术后预后有关的因素（RR=1.584, 95%CI: 1.040-2.411, *P*=0.032）（表 3）。

**3:** 150例NSCLC预后多因素*Cox*回归分析 Survival outcome by multivariate *Cox* proportional hazard analysis in NSCLC (*n*=150)

	B	SE	Wald	df	Sig	Exp (B)	95%CI for Exp (B)	
							Lower	Upper
TNM stage	0.620	0.112	30.896	1	0.000	1.859	1.494	2.314
Methylation status	0.460	0.214	4.598	1	0.032	1.584	1.040	2.411
Age at starting smoking	0.286	0.152	3.525	1	0.060	1.331	0.988	1.795

## 3 讨论

目前已有大量文献报道了在NSCLC、小细胞肺癌、乳腺癌、肾癌、食道癌^[2]^、前列腺癌、胶质瘤^[3]^等多种肿瘤中存在RASSF1A启动子区高甲基化。现已明确抑癌基因*RASSF1A*在肺癌等肿瘤中表达失活的机制是由于启动子区CpG岛的特异性高甲基化所致。虽然抑癌基因的失活也可由突变所致，但对该基因的研究结果显示，RASSF1A的突变率还不及10%，而RASSF1A启动子区发生高甲基化的比率在小细胞肺癌、NSCLC细胞系中分别高达72%-100%、36%-88%，在NSCLC中启动子区高甲基化达30%-45%^[4-7]^，同时非恶性组织肺组织中无一例发生甲基化。在研究中还发现，RASSF1A启动子高甲基化与肺癌肿瘤细胞分化程度、开始吸烟年龄有关^[8, 9]^，本实验研究结果与之相同。这一结果从侧面证实了RASSF1A启动子甲基化状态可能作为预后评价指标，并且早期吸烟可能是造成RASSF1A表观遗传改变的分子事件^[10]^。

在对RASSF1A的大量研究中发现，RASSF1A启动子区甲基化状态极有可能也与肿瘤预后相关。RASSF1A甲基化状态作为肿瘤预后评价的指标在NSCLC中已有证实。Tomizawes等^[11]^在对110例Ⅰ期肺腺癌患者的研究中发现，RASSF1A高甲基化与低生存率相关，并且与肿瘤的低分化程度及血管和胸膜的侵犯高度相关。Kim等^[9]^也发现RASSF1A启动子高甲基化与Ⅰ期、Ⅱ期患者的预后均有关（*P*=0.02, *P*=0.01）。Endoh等^[12]^发现在Ⅰ期和Ⅱ期术后肺癌患者中RASSF1A高甲基化与肺癌术后早期复发相关（*P*=0.024 7）。但是也有1项对116例NSCLC的研究^[13]^发现，RASSF1A的甲基化状态除了与肿瘤分化程度（*P*=0.024）和Ⅰ期肺癌术后生存率（*P*=0.027 6）有关外，与肿瘤的临床特征（TNM分期、肿瘤复发、淋巴转移和是否吸烟）及Ⅱ期和Ⅲ期肺癌术后生存期均无关（*P* > 0.1）。进一步利用*Cox*回归模型分析，结果除了肿瘤分期是肿瘤预后的危险因素外（*P*=0.008 9），其它因素包括RASSF1A甲基化状态在内均与预后无关。因此RASSF1A甲基化并不能作为一个理想的肺癌预后评价指标。鉴于存在不同的研究结果，而国内尚未见RASSF1A异常甲基化在肺癌预后中的文献报道，本实验分析150例NSCLC术后5年生存资料和RASSF1A甲基化状态，发现在Ⅰ期、Ⅱ期和Ⅲa期术后病人中，RASSF1A启动子高甲基化的病例生存率低于未发生甲基化的病例，差异具有统计学意义（*P*=0.022, *P*=0.023, *P*=0.036）。Ⅲb-Ⅳ期的晚期NSCLC患者是否施行原发病灶切除需临床谨慎判断，本实验中也选取了21例Ⅲb-Ⅳ期的原发病灶行手术切除的NSCLC作为研究对象，其中RASSF1A启动子区高甲基化和未甲基化的病例生存期比较无统计学差异（*P*=0.118），考虑由于研究例数较少且晚期肺癌患者后期治疗影响因素较多，结果还有待进一步研究。利用*Cox*模型进行多因素分析，将与NSCLC有关的因素（年龄、性别、组织类型、TNM分期、吸烟指数、开始吸烟年龄等）进行预后相关分析，发现除TNM分期外，RASSF1A启动子区甲基化状态也是影响NSCLC术后生存期的一个独立因素（RR=1.584, 95%CI: 1.040-2.411, *P*=0.032），这一结果与Tomizawa^[11]^和Kim等^[9]^报道结果相同。

*RASSF1A*作为抑癌基因在抑制肿瘤发生中的作用越来越来受到研究者的关注。不断有文献报道，RASSF1A参与细胞周期调节^[14-16]^、诱导细胞凋亡^[17]^、稳定微管^[18, 19]^等多种细胞生理功能。但其使RASSF1A启动子区CpG岛甲基化状态发生变化、引起基因失活的原因尚未完全清楚。本实验在较多人体肺癌组织标本中检测RASSF1A启动子甲基化状态，分析与肺癌临床特征及预后的关系，发现RASSF1A启动子区甲基化状态可以作为NSCLC术后的一个预后评价指标。

## References

[1] Dammann R, Li C, Yoon JH (2000). Epigenetic inactivation of a RAS association domain family protein from the lung tumor suppressor locus 3p21.3. Nat Genet.

[2] Qin YP, Wang LD, Chang ZW (2009). RASSF1A methylation detection in esophageal cancer tissue and matched peripheral blood from the same patients. Chin J Cancer Prev Treat.

[3] Dong XM, Liu YH, Pan WR (2008). The clinical research on promoter methylation of the RASSF1A in glioma. Chin Clini Oncol.

[4] Tsou JA, Shen LYC, Siegmund KD (2005). Distinct DNA methylation profiles in malignant mesothelioma, lung adenocarcinoma, and non-tumor lung. Lung Cancer.

[5] Lin Q, Geng J, Ma K (2009). *RASSF1A* , *APC*, *ESR1*, *ABCB1* and *HOXC9*, but *p16INK4A*, *DAPK1*, *PTEN* and *MT1G* genes were frequently methylated in the stage Ⅰ non-small cell lung cancer in China. J Cancer Res Clin Oncol.

[6] De Jong WK, Verpooten GF, Kramer H (2009). Promoter methylation primarly occurs in tumor cells of patients with non-small cell lung cancer. Anticancer Res.

[7] Divine KK, Pulling LC, Marron-Terada PG (2005). Multiplicity of abnormal promoter methylation in lung adenocarcinomas from smokers and never smokers. Int J Cancer.

[8] Toyooka S, Maruyama R, Toyooka KO (2003). Smoke exposure, histologic type and geography-related differences in the methylation profiles of nonsmall cell lung cancer. Int J Cancer.

[9] Kim DH, Kim JS, Ji YI (2003). Hypermethylation of RASSF1A promoter is associated with the age at starting smoking and a poor prognosis in primary non-small cell lung cancer. Cancer Res.

[10] Marsit CJ, Kim DH, Liu M (2005). Hypermethylation of *RASSF1A* and *BLU* tumour suppressor genes in non-small cell lung cancer: implications for tobacco smoking during adolescence. Int J Cancer.

[11] Tomizawa Y, Kohno T, Kondo H (2002). Clinicopathological signficance of epigentic inactivation of RASSF1A at 3p21.3 in stage Ⅰ lung adenocarcinoma. Clin Cancer Res.

[12] Endoh H, Yatabe Y, Shi mizu S (2003). *RASSF1A* gene inactivation in nonsmall cell lung cancer and its clinical implication. Int J Cancer.

[13] Choei N, Son DS, Song I (2005). RASSF1A is not appropriate as an early detection marker or a prognostic marker for non-small cell lung cancer. Int J Cancer.

[14] Song MS, Chang JS, Song SJ (2005). The centrosomal protein RAS association domain family protein 1A (RASSF1A)-binding protein 1 regulates mitotic progression by recruiting RASSF1A to spindle poles. J Biol Chem.

[15] Song SJ, Song MS, Kim SJ (2009). Aurora A regulates prometaphase progression by inhibiting the ability of RASSF1A to suppress APC-Cdc20 activity. Cancer Res.

[16] Thaler S, Hahnel PS, Schad A (2009). RASSF1A mediates p21Cip1/Waf1-dependent cell cycle arrest and senescence through modulation of the Raf-MEK-ERK pathway and inhibition of Akt. Cancer Res.

[17] Jung H OH, Lee K, Song S J (2006). Role of the tumor suppressor RASSF1A in Mst1-mediated apoptosis. Cancer Res.

[18] van der Weyden L, Tachibana KK, Gonzalez (2005). The RASSF1A isoform of RASSF1A promotes microtubule stability and suppresses tumorigenesis. Mol Cell Biol.

[19] Whang YM, Park KH, Jung HY (2009). Microtubule-damaging agents enhance RASSF1A-induced cell in lung cancer cell lines. Cancer.

